# Home care vehicle routing problem with chargeable overtime and strict and soft preference matching

**DOI:** 10.1007/s10729-020-09532-2

**Published:** 2021-01-22

**Authors:** Laura Malagodi, Ettore Lanzarone, Andrea Matta

**Affiliations:** 1 Department of Mechanical Engineering, Politecnico di Milano, Milan, Italy; 2 Department of Management, Information and Production Engineering, University of Bergamo, Dalmine, (BG) Italy

**Keywords:** Operations research, Home care vehicle routing problem, Chargeable overtime, Preferences, Cluster-based decomposition

## Abstract

A new scheduling problem arising in the home care context is addressed, whose novelty with respect to the literature lies in the way overtime is paid. In this problem, some clients are willing to pay a higher fee to cover the additional overtime cost, if such overtime is incurred because a caregiver works extra time with the client to preserve continuity of care. These overtime hours charged to clients unburden the company, which no longer has to balance between cost and continuity of care in a traditional way. The problem is also studied in a context that includes preferences expressed by both clients and caregivers. Strict preferences must be respected with a high priority, while soft preferences increase the satisfaction and should be preferably respected. We formalize the problem as a Mixed Integer Linear Problem and also propose a cluster-based decomposition to solve real-life instances. The problem is inspired by the real case study of a provider operating in the USA. Numerical results validate the model and confirm the capability of the decomposition approach to deal with real-life instances.

## Highlights

- Chargeable overtime allows home care managers to guarantee continuity of care to clients while avoiding additional overtime cost for the provider.
- The home care scheduling problem is solved by including chargeable overtime and in a context with strict and soft preferences expressed by clients and caregivers.
- A cluster-based decomposition is proposed to solve the problem in real-life instances.
- The problem is inspired by a real provider operating in the USA, but is of general validity for several providers.
- The proposed algorithm allows home care managers to effectively handle the complexity of this scheduling problem in real-life instances.

## Introduction

Home Care (HC) services play an important role in modern health care systems. They can be used in all cases where a patient requires assistance that does not need to be provided in a hospital or a care center. This involves not only medical and nursing assistance, but also cleaning, social assistance and many other activities. The goal is twofold: on the one hand, HC avoids hospitalization cost and, thus, reduces the economic burden on regional and national health care systems; on the other hand, it provides a better quality of life to patients, who are treated at home. HC services are usually provided to elderly frail patients with chronic diseases, or to people with disability. As a consequence, due to aging population, HC services are largely expanding in many Western countries and new and more complex HC facilities are appearing nowadays.

Organizational and planning problems are more complex in HC than in inpatient care, as clients are not in the same location. Thus, in addition to caregivers’ schedules, routing problems and the synchronization with other clients’ activities must also be considered. Additionally, many other constraints must be respected, such as the minimum frequency of visits, caregiver-to-client skill compatibilities, continuity of care, and so on.

Usually, HC management problems can be classified into three planning levels [1, 2]. Middle-term planning (6-24 months) deals with the dimensioning of the HC provider, e.g. the division into districts and the assignment of caregivers, materials and support staff to each district. Short-term planning (1-3 months) deals with the assignment of clients to caregivers, if continuity of care is pursued. Very-short-term planning (1 week) deals with the generation of a weekly plan for each caregiver, including a detailed sequence of visits and tasks. The complexity of all problems increases with the number of clients and caregivers, as well as with the number and structure of regulations and constraints taken into account.

In this article, we address the short-term and very-short-term planning problems for a new and complex class of HC services, inspired by the real case study of a provider operating in the USA. This type of provider takes care of the elderly, who require a variety of services with different characteristics and durations. They range from general household help to clinical and technical services, e.g. injections or qualified assistance in case of specific diseases. Some clients require only few hours a week, while others require ongoing assistance. The novelty of our problem lies in the way overtime is paid and continuity of care is treated.

Continuity of care means that the client is assisted by the least number of caregivers, ideally by a single caregiver who takes care of the entire care or assistance pathway. In our case, some clients are willing to pay a higher fee to cover the additional overtime cost if such overtime is incurred because a caregiver works extra time with the same client to preserve his/her continuity of care.

This additional fee is a fixed amount per hour, given by the additional hourly cost incurred when a caregiver works above the contractual capacity. Each client who is willing to pay it knows the maximum associated cost, which is given by the product between the fee and the number of hours he/she requires. Moreover, a realistic range of variability for the cost is communicated to the client, who decides whether to pay the fee or not. This *chargeable overtime* prevents the company from incurring additional costs deriving from maintaining continuity of care, and from finding a compromise between cost and continuity of care.

The problem is also taking into account preferences. Both clients and caregivers express preferences, classified as strict or soft, and the caregiver-to-client assignments take them into account. Strict preferences must be respected with a high priority, while soft preferences, which increase caregivers’ and clients’ satisfaction and improve the quality of service, should be preferably respected whenever possible.

Although this work is inspired by a real case, the idea of chargeable overtime and the proposed approach can be considered of general validity and extendable to several other HC providers.

Our work consists of two parts. We first formalize the HC assignment and scheduling problem with chargeable overtime and strict and soft preferences, and propose a Mixed Integer Linear Model (MILP) to solve it. The goals are to minimize the non-chargeable overtime paid by the provider, the number of unmatched preferences (distinguishing between strict and soft ones) and the total caregivers’ travel time. Due to the complexity of the model and the difficulty to efficiently tackle real-life instances, we also propose a cluster-based decomposition approach. This separates the problem into independent sub-problems, so that the model can be solved within each cluster and the overall solution is given by the combination of solutions in the clusters. The first part represents the novelty of this work, while the second part shows the applicability of the model in real-life instances.

To validate the model and evaluate the cluster-based decomposition, we have applied our approach to a set of realistic instances derived from the considered real case study, which respect the features of assisted clients and the proportions between each feature in the original data. This allows us to test our approach on several instances and to validate the cluster-based decomposition on larger instances than the current provider needs, to simulate future utilization of the model in a larger context. Results show the effectiveness of proposed formulation and the capability of the cluster-based decomposition to tackle real-life instances in reasonable time.

The rest of the paper is structured as follows. A literature review on related HC problems is presented in Section 2. The addressed problem is described in Section 3 and the mathematical formulation is presented in Section 4. The proposed cluster-based decomposition is detailed in Section 5. The case study is presented in Section 6. The tested instances and the numerical results are reported in Section 7. Finally, the conclusions of the work are drawn in Section 8.

## Literature review

Two literature reviews have recently been published, which provide an overview of HC management problems. [3] published a comprehensive review on HC routing and scheduling with a specific focus on problem setting. [4] identified the most relevant features of HC routing and scheduling problems, analyzed the existing literature based on how the different studies formulate constraints and objective functions, and provided an overview of methods developed to solve the problems.

Several planning problems are involved in HC management, which can be solved either together or separately. [5] divided HC management into three sub-problems, namely grouping, assignment and routing. [6] proposed a three-phase decision support methodology to identify the decision rules for patient acceptance, staff hiring and staff utilization.

HC routing and scheduling problems can be classified based on different characteristics. The main classification is between deterministic and stochastic problems. Another classification concerns the period considered in the formulation, i.e. problems can be single- or multi-period. Finally, widely utilized constraints concern time windows, skill matching, working regulations, and synchronization.

Caregivers have different skills and qualifications, and clients can specify the desire to be assisted by caregivers who meet a specific set of skills. In addition, assignments of caregivers to clients can also be influenced by a number of other characteristics [7–9]. For example, [7] dealt with the penalization of assigning a caregiver with specific skills to a client who does not require them, which is seen as a waste of qualifications.

In some cases, more than one caregiver may be needed to perform a visit. This involves temporal dependencies and the synchronization of caregivers [4, 10]. In the most general case, clients define a time window in which the visit must be performed; within this window, they may also indicate a preferred starting time for the visit. Then, deviations from required time window and preferred start time are usually penalized in the objective function [8, 9].

Overtime and workload fairness are the main metrics for the cost and the quality of work, respectively [11]. Overtime refers to the time worked by a caregiver beyond regular working hours, which is determined by workers’ regulations or by contract. When a caregiver exceeds this threshold, the overtime work is compensated with a higher fee. In models that minimize overtime, the hours above the threshold are penalized in the objective function with a fixed or increasing weight. An upper bound to the number of working hours beyond the threshold can also be added [11, 12]. Workload fairness refers to workload balancing among caregivers [13, 14]. When balancing, either the entire workload amount can be considered, or only one component, e.g. service time or travel time.

Both single-objective and multi-objective problems have been proposed in the literature. In the multi-objective case, most authors optimize a weighted sum of the objectives. Alternatively, [11] proposed a threshold method to include the cost sustained by the provider, workload balancing and continuity of care; [15] proposed a lexicographic approach with two objectives. Finally, [16] determined the entire Pareto frontier using an *ε*-constraint scheme.

In the following paragraphs, we focus on the contributions that are similar to our problem, i.e. research that considers overtime, preferences matching and skill matching.

[7] minimized travel time, dissatisfied clients, overtime and visits to clients who require a lower qualification level, and included preferred time windows for both caregivers and clients. However, dissatisfaction simply considers the possibility for clients to ask for a specific caregiver. The total overtime per caregiver is considered, and there is no possibility for the client to pay it. [17] minimized the cost of each caregiver, given by service and travel times, and maximized clients’ and caregivers’ satisfaction. They included additional tasks for caregivers (e.g. team meetings) and the fact that patients and caregivers may declare negative preferences against each other. Finally, the authors took multi-modality into account, i.e. different modes of transport. They did not consider overtime. [18] developed a heuristic approach to minimize the total travel time, the total idle time due to early arrivals with respect to the target time, and the remaining time until the end of the working time window. They penalized task and time window violations, working time violations, skill requirement violations, assignments of non-preferred tasks and caregivers, connected task violations. However, they minimized overtime without the possibility of charging it to clients and based preferences on skill requirements only. Braekers et al. [16] developed a metaheuristic algorithm in which clients specify preferences for caregivers through a penalty incurred when assigning a certain caregiver to a specific visit. However, this penalty is simply related to the caregiver and not based on a set of criteria as in our case. The total number of worked hours was considered to compute the overtime, neglecting the possibility of clients paying for it. Finally, [9] minimized total time, uncovered visits, overtime work and deviations from the preferred time of jobs.

To the best of our knowledge, no available contributions consider the idea of chargeable overtime as this work does. Regarding the matching criteria, none of the available contributions include a rigid classification with several criteria and two levels (strict and soft preferences) as considered in our problem.

We conclude our literature overview exploring similar contributions in other fields, to look for chargeable overtime in other applications or similar matching criteria problems. In fact, our problem can be seen as an extension of both the Vehicle Routing Problem (VRP) and the Nurse Rostering Problem (NRP), for which a huge literature with many variants is available. Our problem can be considered as a VRP with time windows and skills [19], if we extend the idea of skill to account for both strict and soft preferences. To the best of our knowledge, the possibility of charging the requested overtime to the client cannot be found in the VRP literature. The NRP deals with scheduling employees’ shifts for inpatient institutions, e.g. residential homes and hospitals. Preferences and skill compatibilities are sometimes considered; however, travel time is not included due to the inpatient institution. In this case as well, chargeable overtime cannot be found in the NRP literature.

Looking at matching problems, they are widely studied in several fields. Considering health care related problems, a relevant case is the so-called Hospitals/Residents Problem [20]. Outside the health care field, a matching structure close to that considered in our work can be found in the Stable Marriage problem [21], whose goal is to find a stable matching between two sets of individuals given an ordering of preferences for each individual. However, matching problems neglect all other time-related features that are typical of a scheduling problem, e.g. workloads and travel times.

This short analysis confirms the novelty of our problem, especially in regards to the chargeable overtime.

## Problem description

The addressed problem includes both classical features of assignment and scheduling problems and the new features considered in our work, i.e. the chargeable overtime coupled with the matching criteria.

The problem consists of assigning a set *I* of jobs required by a set *C* of clients to a set *K* of caregivers over a planning horizon divided into a set *H* of discrete time periods. Parameters ${\theta _{i}^{c}}$ denote the correspondence between jobs and clients, i.e. ${\theta _{i}^{c}}=1$ if job *i* ∈ *I* belongs to client *c* ∈ *C* and 0 otherwise (with ${\sum }_{c \in C} {\theta _{i}^{c}} = 1$ ∀*i* ∈ *I*). Each job *i* ∈ *I* is characterized by a fixed duration *d*_*i*_ and a given starting time *t*_*i*_ ∈ *H*, and requires only one caregiver. Some time periods refer to the weekend (subset *W* ⊂ *H*) and some others to the night (subset *N* ⊂ *H*); a job *i* is considered to be a job at night if *t*_*i*_ ∈ *N* or a job in the weekend if *t*_*i*_ ∈ *W*.

Each caregiver *k* ∈ *K* begins and ends the route in his/her own depot, while each client *c* ∈ *C* is located at his/her domicile. We denote the travel time from job *i* ∈ *I* to *j* ∈ *I* or vice versa (between the respective clients’ domiciles) by *δ*_*ij*_, while the travel time from the depot of caregiver *k* ∈ *K* to job *i* ∈ *I* or vice versa by $\widetilde {\delta }_{i}^{k}$.

Each caregiver *k* ∈ *K* has a maximum working time over *H*, denoted by *S*_*k*_. This amount is given by the regular working time *τ* reported in the working contract (without loss of generality, *τ* is assumed to be the same for all caregivers) and the maximum overtime the caregiver is willing to work. Though travel times are minimized in the problem, caregivers’ workload refers only to the service time with clients, as is the case with several HC providers including the one considered in this work.

Finally, the problem is characterized by chargeable overtime, preference matching, and specific characteristics of jobs and shifts, which are separately presented below.

### Chargeable overtime

The overtime of caregiver *k* ∈ *K* is given by the amount of time that *k* works beyond his/her regular working time *τ*. As overtime hours are paid with a higher fee, it is not profitable for the provider to exploit overtime. In addition, overloaded caregivers might provide lower quality service.

Avoiding overtime is in contrast with favoring continuity of care, and providers usually assign multiple caregivers to clients to remove or reduce overtime, though this deteriorates continuity of care. This trade-off between overtime reduction and continuity of care is particularly critical in several new and complex HC facilities where clients ask for a high number of hours, as in the considered case study. To solve the trade-off, the considered provider introduced the idea of chargeable overtime. Each client can choose whether or not to pay a higher fee to cover the additional overtime costs if these costs are incurred for providing the visits to this client. For clients willing to pay the additional fee, no additional costs are incurred by the provider and continuity of care is pursued with no drawbacks. In the case of clients who are not willing to pay the additional fee, continuity of care could be affected more, since the provider minimizes overtime. This idea is of general validity and can be effectively applied to other HC providers; from an academic viewpoint, it is novel in the HC management literature.

A binary parameter *ϕ*_*c*_ is associated with each client *c* ∈ *C*, which is equal to 1 if *c* is willing to pay the additional fee and 0 otherwise. Then, the total overtime of each caregiver *k* ∈ *K* is split into two parts and only the part *σ*_*k*_ (decision variable) not paid by clients with *ϕ*_*c*_ = 1 is minimized. In any case, the maximum working time *S*_*k*_ of each caregiver *k* is respected.

### Preferences

The provider takes into account a list of preferences that may impact quality and satisfaction of caregiver-client matching. Each preference is expressed by the client in terms of a yes/no requirement, i.e. the client may require a caregiver with or without a specific characteristic. Preferences include caregiver’s gender and other personal characteristics, e.g. the caregiver being a smoker or not. Based on their relevance, these preferences are divided into a set *M* of strict preferences, which must be respected with a high priority, and a set *F* of soft preferences, which should be preferably respected.

Each client *c* ∈ *C* expresses his/her interest for each characteristic. Parameter ${\lambda _{c}^{q}}$ is equal to 1 if client *c* expresses interest in the strict preference *q* ∈ *M* and 0 if he/she is indifferent to *q*; parameter ${\mu _{c}^{r}}$ is equal to 1 if client *c* expresses interest in the soft preference *r* ∈ *F* and 0 if he/she is indifferent to *r*.

For each strict preference *q* ∈ *M*, parameter ${\omega _{k}^{q}}$ is equal to 1 if caregiver *k* ∈ *K* has the considered characteristic and 0 otherwise. Parameter ${\pi _{c}^{q}}$ is equal to 1 if client *c* ∈ *C* wants a caregiver with this characteristic and 0 if he/she wants a caregiver without this characteristic (considered only if ${\lambda _{c}^{q}}=1$).

Similarly, for each soft preference *r* ∈ *F*, parameter ${\psi _{k}^{r}}$ is equal to 1 if caregiver *k* ∈ *K* has the considered characteristic and 0 otherwise. Parameter ${\chi _{c}^{r}}$ is equal to 1 if client *c* ∈ *C* wants a caregiver with this characteristic and 0 if he/she wants a caregiver without this characteristic (considered only if ${\mu _{c}^{r}}=1$).

With these parameters we can compute ${\gamma _{c}^{k}}$ and $\widetilde {\gamma }_{c}^{k}$ that express the number of total strict and soft preference mismatches between *c* ∈ *C* and *k* ∈ *K*, respectively, as follows:

1 $$ \begin{array}{@{}rcl@{}} && {\gamma^{k}_{c}} = {\sum}_{q \in M} \left( {\pi_{c}^{q}} - {\omega_{k}^{q}} \right)^{2} {\lambda_{c}^{q}} \qquad\qquad\quad \forall c \in C, k \in K \end{array} $$

2 $$ \begin{array}{@{}rcl@{}} && \widetilde{\gamma}^{k}_{c} = {\sum}_{r \in F} \left( {\chi_{c}^{r}} - {\psi_{k}^{r}} \right)^{2} {\mu_{c}^{r}} \qquad\qquad\quad \forall c \in C, k \in K \end{array} $$

The calculations within parentheses are equal to 1 if there is a mismatch between *c* and *k* for the considered preference, while the multiplication by ${\lambda _{c}^{q}}$ or ${\mu _{c}^{r}}$ allows to compute the mismatches for the items of interest only.

The two sets of parameters ${\gamma _{c}^{k}}$ and $\widetilde {\gamma }_{c}^{k}$ are included in the model.

### Characteristics of jobs and shifts

Besides chargeable overtime and preference matching, which are the novel features considered together in this work, we tailor the characteristics of jobs and shifts on the considered case study.

The time horizon includes nights and weekends, and each caregiver decides whether he/she is willing to work in these time frames. Parameter *ξ*_*k*_ is equal to 1 if caregiver *k* is willing to work on the weekend and 0 otherwise, while parameter *ν*_*k*_ is equal to 1 if caregiver *k* is willing to work at night and 0 otherwise.

In any case, each caregiver must return home for at least *ρ* every *D* time periods, for a home-based break. From a practical viewpoint *D* represents the number of time periods in a day (e.g. *D* = 24 with periods of 1 hour) to impose that each caregiver has his/her own daily home-based break. On the contrary, when the caregiver does not return home between two jobs, the maximum time that he/she can spend in a break is limited to β time periods.

One of the main features of the problem, inspired by the considered provider, is the so-called *night shift*. These shifts cover the whole night but, if the client does not require continuous assistance overnight, then the caregiver can rest and sleep at the client’s home for a period of *ρ* time periods. In this way, if the client is willing to host a caregiver for the night, travel time and the caregiver’s discomfort are avoided. These hours of rest are not considered as working hours, they are not paid (neither by the client nor by the provider) and do not contribute to caregivers’ workloads. This night shift arrangement is identified by a parameter *n*_*i*_ for each job: *n*_*i*_ = 1 if job *i* ∈ *I* is a night shift and 0 otherwise. If *n*_*i*_ = 1 the duration *d*_*i*_ does not include the *ρ* periods of rest, to take into account that they are not paid.

Night shifts can only be assigned to caregivers who are willing to stay overnight. Parameter *λ*_*k*_ is equal to 1 if caregiver *k* is willing to perform night shifts and 0 otherwise (*λ*_*k*_ = 1 only if *ν*_*k*_ = 1). When a caregiver rests at a client’s home, this rest period replaces the home-based break of at least *ρ* periods.

## Mathematical formulation

The assignment of jobs to caregivers is modeled by binary variables ${z_{i}^{k}}$, where ${z_{i}^{k}} = 1$ if job *i* ∈ *I* is assigned to caregiver *k* ∈ *K* and 0 otherwise.

The goals of the assignment are to minimize the non-chargeable overtime paid by the provider, the number of unmatched preferences (distinguishing between strict and soft) and the total caregivers’ travel time.

Set, parameters and decision variables are listed in Table 1, while the MILP formulation is presented below.

**Table 1:** Set, parameters and decision variables of the MILP model

Sets	
*I*	set of jobs
*C*	set of clients
*K*	set of caregivers
*M*	set of strict preferences
*F*	set of soft preferences
*H*	set of time periods in the planning horizon
*W* ⊂ *H*	subset of weekend time periods
*N* ⊂ *H*	subset of night time periods
Parameters	
*d*_*i*_	duration of job *i* ∈ *I* in time periods
*t*_*i*_	starting time period of job *i* ∈ *I*
${\theta _{i}^{c}}$	equal to 1 if job *i* ∈ *I* belongs to client *c* ∈ *C*; 0 otherwise
*n*_*i*_	equal to 1 if job *i* ∈ *I* requires a night shift afterwards; 0 otherwise
*τ*	regular working time (same for all caregivers *k* ∈ *K*)
*S*_*k*_	maximum working time of caregiver *k* ∈ *K*
*ϕ*_*c*_	equal to 1 if client *c* ∈ *C* is willing to pay overtime; 0 otherwise
${\gamma _{c}^{k}}$	number of strict preference mismatches between client *c* ∈ *C* and caregiver *k* ∈ *K*
$\widetilde {\gamma }_{c}^{k}$	number of soft preference mismatches between client *c* ∈ *C* and caregiver *k* ∈ *K*
*δ*_*ij*_	travel time from job *i* ∈ *I* to job *j* ∈ *I* or vice versa
$\widetilde {\delta }_{i}^{k}$	travel time from depot of caregiver *k* ∈ *K* to job *i* ∈ *I* or vice versa
*ρ*	minimum number of time periods a caregiver can spend on a home-based break
β	maximum number of time periods a caregiver can spend on a break without going home
*ξ*_*k*_	equal to 1 if caregiver *k* ∈ *K* is willing to work on the weekend; 0 otherwise
*ν*_*k*_	equal to 1 if caregiver *k* ∈ *K* is willing to work at night; 0 otherwise
*λ*_*k*_	equal to 1 if caregiver *k* ∈ *K* is willing to perform night shifts; 0 otherwise
*D*	number of time periods in a day
Λ	big number
*α*_*M*_	weight of the strict preference mismatch in the objective function
*α*_*F*_	weight of the soft preference mismatch in the objective function
*α*_*O*_	weight of the overtime paid by the provider in the objective function
*α*_*T*_	weight of the total travel time in the objective function
Decision variables	
${z^{k}_{i}}$	equal to 1 if job *i* ∈ *I* is assigned to caregiver *k* ∈ *K*; 0 otherwise
$x^{k}_{ij} $	equal to 1 if job *j* ∈ *I* is done immediately after job *i* ∈ *I* by caregiver *k* ∈ *K*
	without returning home; 0 otherwise
$y^{k}_{ij} $	equal to 1 if job *j* ∈ *I* is done immediately after job *i* ∈ *I* by caregiver *k* ∈ *K*
	when returning home between the two jobs; 0 otherwise
${f_{i}^{k}}$	equal to 1 if job *i* ∈ *I* is the first job of caregiver *k* ∈ *K*; 0 otherwise
${l_{i}^{k}}$	equal to 1 if job *i* ∈ *I* is the last job of caregiver *k* ∈ *K*; 0 otherwise
${W^{k}_{c}}$	overtime (positive) or undertime (negative) of caregiver *k* ∈ *K* on client *c* ∈ *C*
${O^{k}_{c}} $	overtime of caregiver *k* ∈ *K* on client *c* ∈ *C*
*σ*_*k*_	overtime of caregiver *k* ∈ *K* not paid by the clients
*u*	auxiliary binary variable
${v^{k}_{c}}$, ${\omega ^{k}_{c}}$	auxiliary binary variables ∀*c* ∈ *C*,*k* ∈ *K*
*χ*_*ij*_	auxiliary binary variables ∀*i*,*j* ∈ *I*

The model minimizes the following weighted sum:

3 $$ \begin{array}{@{}rcl@{}} \text{minimize} && \left\{ \alpha_{M} {\sum}_{\substack{i \in I \\ c \in C \\ k \in K}} d_{i} {\gamma_{c}^{k}} {\theta_{i}^{c}} {z^{k}_{i}} + \alpha_{F} {\sum}_{\substack{i \in I \\ c \in C \\ k \in K}} d_{i} \widetilde{\gamma}_{c}^{k} {\theta_{i}^{c}} {z^{k}_{i}} + \alpha_{O} {\sum}_{k \in K} \sigma_{k}\right. \\ && \left. + \alpha_{T} \left[ {\sum}_{\substack{i \in I \\ k \in K}} {\widetilde\delta^{k}_{i}} {f^{k}_{i}} + {\sum}_{\substack{i \in I \\ k \in K}} {\widetilde\delta^{k}_{i}} {l^{k}_{i}} + {\sum}_{\substack{i \in I \\ j \in J \\ k \in K}} \delta_{ij} x^{k}_{ij} + {\sum}_{\substack{i \in I \\ j \in J \\ k \in K}} ({\widetilde\delta^{k}_{i}} + {\widetilde\delta^{k}_{j}}) y^{k}_{ij} \right] \right\} \end{array} $$

The first two terms (weighted by *α*_*M*_ and *α*_*F*_, respectively) compute the mismatches of strict and soft preferences over the jobs. Both are multiplied by the duration *d*_*i*_ of the job, to penalize mismatches on longer-lasting jobs more. The third term (weighted by *α*_*O*_) is the total overtime paid by the provider and not by clients. The last four terms (all weighted by *α*_*T*_) give the total travel time for all caregivers *k* ∈ *K*. The first term is the total time that the caregivers travel from the depot to the first job; the second the total time that the caregivers travel from the last job to the depot; the third the total time that the caregivers travel to directly go from a job to the next one; the fourth the total time that the caregivers travel between two jobs when they go home in between. The decision variables ${f_{i}^{k}}$ are equal to 1 if *i* is the first job of caregiver *k* and 0 otherwise; decision variables ${l_{i}^{k}}$ are equal to 1 if *i* is the last job of caregiver *k* and 0 otherwise. The main objective is to minimize the mismatches of strict preferences. Thus, *α*_*M*_ >> *α*_*F*_ ≈ *α*_*O*_ ≈ *α*_*T*_ to penalize the mismatches of strict preferences more.

The first group of constraints regulates the assignments of jobs:

4 $$ \begin{array}{@{}rcl@{}} && {\sum}_{k\in K} {z^{k}_{i}} = 1 \qquad\qquad\qquad\qquad\qquad\qquad \forall i \in I \end{array} $$

5 $$ \begin{array}{@{}rcl@{}} && {\sum}_{i \in I} d_{i} {z^{k}_{i}} \leq S_{k} \qquad\qquad\qquad \qquad\qquad \forall k \in K \end{array} $$

6 $$ \begin{array}{@{}rcl@{}} && {\sum}_{i \in I:  t_{i} \in W} {z^{k}_{i}} \leq {\Lambda}  \xi_{k} \qquad\qquad\qquad \qquad  \forall k \in K \end{array} $$

7 $$ \begin{array}{@{}rcl@{}} && {\sum}_{i \in I:  t_{i} \in N} {z^{k}_{i}} \leq {\Lambda}  \nu_{k} \qquad\qquad\qquad \qquad  \forall k \in K \end{array} $$

8 $$ \begin{array}{@{}rcl@{}} && n_{i} {z^{k}_{i}} \le \lambda_{k} \qquad\qquad\qquad \qquad   \forall i \in I, k \in K \end{array} $$

Constraints (4) guarantee that each job is assigned to exactly one caregiver. Constraints (5) limit the total working time for each caregiver *k*, which can be at maximum *S*_*k*_. Constraints (6) and (7) guarantee that, if a caregiver is not available at night or on the weekend, no jobs are assigned during this time period (Λ is a big number, both here and below). Constraints (8) guarantee that night shifts are assigned only to caregivers willing to perform them.

The second group guarantees that the starting times of jobs are correctly sequenced:

9 $$ \begin{array}{@{}rcl@{}} & t_{i} \!+ d_{i} \!+ \rho  n_{i}\! + \delta_{ij}\! -\! {\Lambda}\! \left( \! 1 - x^{k}_{ij} \right)\! \leq t_{j} \qquad\quad \forall i,j\! \in I, k\! \in K \end{array} $$

10 $$ \begin{array}{@{}rcl@{}} & t_{i}\! + d_{i}\! + \rho  n_{i} \!+ \widetilde {\delta^{k}_{i}} \!+ {\widetilde\delta^{k}_{j}} - {\Lambda}\! \left( \! 1 - y^{k}_{ij} \right)\! \leq t_{j} \quad \forall i,j \in I, k \in K \end{array} $$

Constraints (9) state that, if job *j* is performed immediately after *i* by the same caregiver *k*, the starting time of *j* cannot be before the completion time of *i* plus the travel time from *i* to *j*. Similarly, constraints (10) consider the travel time to go from job *i* to the caregiver’s home and from there to job *j*. In both cases, the completion time of job *i* is given by *d*_*i*_ + *ρn*_*i*_ to include the additional *ρ* rest hours spent at the client’s home in case of night shift (when *n*_*i*_ = 1). Decision variables $x^{k}_{ij}$ are equal to 1 if job *j* is done immediately after *i* by caregiver *k* without returning home, and 0 otherwise; decision variables $y^{k}_{ij}$ are equal to 1 if job *j* is done immediately after *i* by caregiver *k* when returning home between the two jobs, and 0 otherwise.

The third group gives the relations between the assignment variables ${z^{k}_{i}}$, $x^{k}_{ij}$, $y^{k}_{ij}$, ${f_{i}^{k}}$ and ${l_{i}^{k}}$:

11 $$ \begin{array}{@{}rcl@{}} && {\sum}_{i \in I } \left( x^{k}_{ij} + y^{k}_{ij} \right) + {f^{k}_{j}} - {z^{k}_{j}} = 0 \qquad\quad \forall j \in I, k \in K \end{array} $$

12 $$ \begin{array}{@{}rcl@{}} && {\sum}_{j \in j} \left( x^{k}_{ij}+ y^{k}_{ij} \right) + {l^{k}_{i}} - z^{k}_ i= 0 \qquad\quad \ \ \forall i \in I, k \in K \end{array} $$

13 $$ \begin{array}{@{}rcl@{}} && {\sum}_{i\in I} {z^{k}_{i}} \leq {\Lambda} {\sum}_{i\in I} {f^{k}_{i}} \qquad\qquad\qquad ~~~~~~~~~~~~~~~~~~\forall k\in K \end{array} $$

14 $$ \begin{array}{@{}rcl@{}} && {\sum}_{i \in I} {z^{k}_{i}} \geq {\sum}_{i \in I} {f^{k}_{i}} \qquad\qquad\qquad ~~~~~~~~~~~~~~~~~~~~~~\forall k \in K \end{array} $$

15 $$ \begin{array}{@{}rcl@{}} && {\sum}_{i \in I} {f^{k}_{i}} \leq 1 \qquad\qquad\qquad ~~~~~~~~~~~~~~~~~~~~~~~~~~~~~~\forall k\in K \end{array} $$

16 $$ \begin{array}{@{}rcl@{}} && {\sum}_{i\in I} {z^{k}_{i}} \leq {\Lambda} {\sum}_{i\in I} {l^{k}_{i}} \qquad\qquad\qquad ~~~~~~~~~~~~~~~~~~~~\forall k \in K \end{array} $$

17 $$ \begin{array}{@{}rcl@{}} && {\sum}_{i \in I} {z^{k}_{i}} \geq {\sum}_{i \in I} {l^{k}_{i}} \qquad\qquad\qquad ~~~~~~~~~~~~~~~~~~~~~~~~\forall k \in K \end{array} $$

18 $$ \begin{array}{@{}rcl@{}} && {\sum}_{i \in I} {l^{k}_{i}} \leq 1 \qquad\qquad\qquad ~~~~~~~~~~~~~~~~~~~~~~~~~~~~~~~~\forall k \in K \end{array} $$

19 $$ \begin{array}{@{}rcl@{}} && x^{k}_{ij} + y^{k}_{ij} \leq 1 \qquad\qquad\qquad ~~~~~~~~~~~~~\forall i,j \in I, k \in K \end{array} $$

20 $$ \begin{array}{@{}rcl@{}} && x^{k}_{ii} = 0 \qquad\qquad\qquad ~~~~~~~~~~~~~~~~~~~~~~~~~~\forall i \in I, k \in K \end{array} $$

21 $$ \begin{array}{@{}rcl@{}} && y^{k}_{ii} = 0 \qquad\qquad\qquad ~~~~~~~~~~~~~~~~~~~~~~~~~~\forall i \in I, k \in K \end{array} $$

Constraints (11) guarantee that, if caregiver *k* does job *j*, this job can be either the first or the successor of another job *i* (returning home after *i* or not). Similarly, constraints (12) guarantee that, if caregiver *k* does job *i*, this job can be either the last or the predecessor of another job *j* (returning home before *j* or not). Constraints (13)-(15) regulate the first job ${f^{k}_{i}}$: constraints (13) guarantee that, if caregiver *k* performs at least one job, there is a first job among those performed; constraints (14) guarantee that, if caregiver *k* has a first job, he/she has at least one job assigned; constraint (15) impose that a caregiver cannot have more than one first job. Constraints (16)-(18) regulate the last job ${l^{k}_{i}}$ in the same way. Constraints (19) impose that, when a job is done immediately after another, either the caregiver returns home or not between the jobs. Finally, Eqs. 20 and 21 are consistency constraints on $x^{k}_{ij}$ and $y^{k}_{ij}$.

The fourth group guarantees that, if the time between two jobs is greater than β, the caregiver goes home:

22 $$ \begin{array}{@{}rcl@{}} && t_{j} - \left( t_{i} + d_{i} + \rho  n_{i} + \upbeta \right) \le {\Lambda} \chi_{ij} \qquad\quad \forall i,j \in I \end{array} $$

23 $$ \begin{array}{@{}rcl@{}} && {\sum}_{k \in K} x^{k}_{ij} \le {\Lambda} \left( 1 - \chi_{ij} \right) \qquad\qquad\qquad ~~~~~\forall i,j \in I \end{array} $$

Constraints (22) impose that the auxiliary variables *χ*_*ij*_ are equal to 1 if the time between the end of job *i* and the beginning of job *j* is equal to or greater than β. Then, constraints (23) impose $x^{k}_{ij}=0$ for all caregivers *k* if *χ*_*ij*_ = 1, i.e. that job *j* cannot be done immediately after *i* without going home.

The fifth group computes the overtime:

24 $$ \begin{array}{@{}rcl@{}} && {\sum}_{i \in I} {z^{k}_{i}} d_{i} {\theta_{i}^{c}} = \tau + {W^{k}_{c}} \qquad \qquad  ~~~~~~~~\forall c \in C, k \in K \end{array} $$

25 $$ \begin{array}{@{}rcl@{}} && {W^{k}_{c}} \leq {\Lambda} {v^{k}_{c}} \qquad \qquad \qquad ~~~~~~~~~~~~~~~~~\forall c \in C, k \in K \end{array} $$

26 $$ \begin{array}{@{}rcl@{}} && {O^{k}_{c}}- {W^{k}_{c}} \leq {\Lambda} \left( 1-{v^{k}_{c}} \right) ~~\quad \quad ~~~~~~~~\forall c \in C, k \in K \end{array} $$

27 $$ \begin{array}{@{}rcl@{}} && - {W^{k}_{c}} \leq {\Lambda} {\omega^{k}_{c}} \qquad \qquad \qquad ~~~~~~~~~~~~~\forall c \in C, k \in K \end{array} $$

28 $$ \begin{array}{@{}rcl@{}} && {O^{k}_{c}} \leq {\Lambda} \left( 1-{\omega^{k}_{c}} \right) \qquad \qquad \qquad ~~~~\forall c \in C, k \in K \end{array} $$

29 $$ \begin{array}{@{}rcl@{}} && {\sum}_{\substack{i \in I \\ c \in C}} z^{k}_{i }d_{i} {\theta_{i}^{c}} \leq \tau + \sigma_{k} + {\sum}_{c \in C} {O^{k}_{c}} \phi_{c} \qquad \qquad \forall k \in K \end{array} $$

Constraints (24) define the total working time ${W^{k}_{c}}$ that each caregiver *k* works on each client *c* above or below *τ* (${W^{k}_{c}}>0$ for overtime above *τ*; ${W^{k}_{c}}<0$ for undertime below *τ*). Constraints (25)-(28) compute the overtime ${O^{k}_{c}}$ of caregiver *k* devoted to client *c*. If ${W^{k}_{c}}>0$, constraints (25) impose that each auxiliary binary variable ${v^{k}_{c}}$ is equal to 1; thus, ${O^{K}_{c}} \leq {W^{k}_{c}}$ from constraints (26). If ${W^{k}_{c}}<0$, no restriction to the value of ${O^{k}_{c}}$ is imposed by these two constraints, while constraints (27) impose that each auxiliary variable ${\omega ^{k}_{c}}$ is positive; thus, ${O^{k}_{c}}=0$ from constraints (28) and the domain ${O^{k}_{c}} \ge 0$. Finally, constraints (29) compute *σ*_*k*_ based on ${O^{k}_{c}}$ and the willingness of clients to pay overtime *ϕ*_*c*_.

Finally, the last constraints define the domains of decision variables:

$$ \begin{array}{@{}rcl@{}} && {O^{k}_{c}} \in \mathbb{N} \qquad\qquad\qquad ~~~~~~~~~~~~~~~~~~~~~\forall c \in C, k\in K \\ && {W^{k}_{c}} \in \mathbb{Z} \qquad\qquad\qquad ~~~~~~~~~~~~~~~~~~~~~\forall c \in C, k\in K \\ && \sigma_{k} \in \mathbb{N} \qquad\qquad \qquad~~~~~~~~~~~~~~~~~~~~~~~~~~~~~~~~~~\forall k \in K \\ && {z^{k}_{i}}, {f^{k}_{i}}, {l^{k}_{i}} \in \{0,1\} \qquad\qquad\qquad ~~~~~~~\forall i \in I, k \in K \\ && x^{k}_{ij}, y^{k}_{ij} \in \{0,1\} \qquad\qquad\qquad ~~~~~~~\forall i,j \in I, k\in K \\ && u \in \{0,1\} \\ && {v^{k}_{c}}, {\omega^{k}_{c}} \in \{0,1\} \qquad\qquad\qquad ~~~~~~~~~~\forall c \in C, k\in K \\ && \chi_{ij} \in \{0,1\} \qquad\qquad\qquad ~~~~~~~~~~~~~~~~~~~~~~~~\forall i,j \in I \end{array} $$

Continuity of care is inherently pursued, because the minimization of preference violations and travel time favors a single caregiver rather than several ones if this solution does not affect the cost sustained by the provider. However, if the number of hours requested by a client is high, this could result in several caregivers due to the extra overtime cost that the provider should pay if a single caregiver provides all visits to the client. The chargeable overtime avoids such extra cost and allows the model to pursue continuity of care for clients who are willing to pay the extra fee. However, in some cases, this effect can be prevented by non-consecutive jobs from the same client, too many jobs from the same client, too long jobs, or a high percentage of clients with *ϕ*_*c*_ = 1. In these cases, to enforce the continuity of care requirement, the reduction of the number of caregivers assigned to each client with *ϕ*_*c*_ = 1 can be explicitly pushed. To this end, the following term is added to the weighted sum minimized in the objective function:

30 $$ \alpha^{*} {\sum}_{\substack{c \in C \\ k \in K}} \phi_{c} {p_{c}^{k}}  $$

where *α*^∗^ denotes the weight of this term, each ${p_{c}^{k}}$ is a binary decision variable equal to 1 if at least one job of client *c* ∈ *C* is assigned to caregiver *k* ∈ *K*, and 0 otherwise, and ${\sum }_{k \in K} {p_{c}^{k}}$ computes the number of caregivers assigned to client *c* ∈ *C*, whose sum over the clients willing to pay overtime is minimized. The new variables ${p_{c}^{k}}$ are computed through the following constraints:

31 $$ \begin{array}{@{}rcl@{}} && {\Lambda}  {p_{c}^{k}} \ge {\sum}_{i \in I} {\theta_{i}^{c}} {z_{i}^{k}} \qquad\qquad\qquad \forall c \in C, k\in K \end{array} $$

32 $$ \begin{array}{@{}rcl@{}} && {p_{c}^{k}} \le {\sum}_{i \in I} {\theta_{i}^{c}} {z_{i}^{k}} \qquad\qquad\qquad ~~~~\forall c \in C, k\in K \end{array} $$

## Cluster-based decomposition

The model presented in Section 4 cannot be efficiently solved in larger instances. Thus, a decomposition of the problem into sub-problems and/or a heuristic approach is required to apply the tool in the case of large providers. In this work, we follow the first alternative to preserve the exact structure of the model. The problem is decomposed into independent sub-problems that are separately solved, and the solution of the overall problem is the combination of solutions in the sub-problems. The final solution is clearly sub-optimal, but with a significant advantage in terms of computational performance.

The adopted two-step algorithm first divides the caregivers into a set *L* of clusters, and then adds the clients to these clusters. Finally, if some clusters remain without clients, their caregivers are redistributed to the other clusters that contain clients. As the main objective is to minimize the mismatches of strict preferences (*α*_*M*_ significantly greater than the other weights), the clustering is performed by grouping together the caregivers *k* ∈ *K* whose vectors ${\Omega }_{k} = \left [ {\omega _{k}^{q}}, q \in M \right ]$ assume similar values (see Section 3.2).

The clusters are generated by means of a hierarchical clustering [22], adopting a Manhattan distance and a complete linkage over the vectors of strict preferences Ω_*k*_. The number of clusters |*L*| is obtained by imposing the cut-off point Γ in the dendrogram of the hierarchical clustering. Then, the assignment of clients to clusters is performed with the *k*-Nearest Neighbors (*k* − *NN*) algorithm [23].

Suitable values for the cut-off point Γ and the parameter *k* of the *k* − *NN* algorithm are not known *a priori*, but they must be chosen based on numerical experiments. In fact, the best values are those that provide the lowest objective function for the problem rather than good clustering metrics.

It is important to notice that this two-step algorithm may lead to an infeasible solution for a sub-problem. In some cases, it is necessary to solve the sub-problem to detect the infeasibility. In other cases, the infeasibility can be detected in advance without solving the sub-problem. Thus, the algorithm also includes a detection of the infeasibilities that can be identified before solving the sub-problems. This is done by considering the total demand from clients and the total availability of caregivers. More specifically, we evaluate the following demand-to-workload ratio in each cluster *l* ∈ *L*:

$$ r^{(l)} = \frac{{\sum}_{\substack{i \in I \\ c \in C^{(l)}}} d_{i} {\theta_{i}^{c}}}{{\sum}_{k \in K^{(l)}}{S_{k}}} $$

where *C*^(*l*)^ and *K*^(*l*)^ denote the subsets of clients and caregivers assigned to cluster *l*, respectively. We impose that *r*^(*l*)^ ≤ *η* ∀*l* ∈ *L*, where *η* is a predefined threshold. Obviously, *η* ≤ 1 because *r*^(*l*)^ > 1 certainly provides an infeasible problem, while *r*^(*l*)^ ≤ 1 could admit a feasible solution and the chance to find a feasible solution increases as *r*^(*l*)^ decreases. In this regard, it is worth remarking that, even when *r*^(*l*)^ = 1, the problem could be infeasible because some jobs cannot be allocated to the caregivers, and the duration of these non-assigned jobs equals the non-used capacity of caregivers.

Operatively, the two-step algorithm (hierarchical clustering and *k* − *NN*) is first executed as described above. Then, if one or more clusters do not respect the condition *r*^(*l*)^ ≤ *η*, the algorithm is reiterated with the inclusion of some adjustments until such condition is respected ∀*l* ∈ *L*. Adjustments consist of moving clients from an overloaded cluster *l* with *r*^(*l*)^ > *η* to another one with *r*^(*l*)^ ≤ *η* following these steps:

1. The similarity between each client and the set of caregivers in the cluster is measured. This measure considers the same criterion that initially composes the clusters, i.e. the closeness of vector ${\Pi }_{c} = \left [ {\pi _{c}^{q}}, q \in M \right ]$ of the considered client *c* with respect to the vectors ${\Omega }_{k} = \left [ {\omega _{k}^{q}}, q \in M \right ]$ of caregivers *k* in the cluster, evaluating only the components for which ${\lambda _{c}^{q}}=1$. According to this, we define the dissatisfaction of client *i* with being in cluster *l* as:
  $$dis_{c}^{(l)} = \frac{{\sum}_{\substack{k \in K^{(l)} \\ q \in M}}{{\lambda_{c}^{q}} \left| {\pi_{c}^{q}} -{\omega_{k}^{q}} \right|}}{{\sum}_{q \in M} {\lambda_{c}^{q}}}$$
2. The clients in each overloaded cluster *l* ∈ *L* are sorted based on their dissatisfaction index, and the client with the highest value of the index is removed. If this removal is sufficient to respect *r*^(*l*)^ ≤ *η* the removal in cluster *l* is stopped. Otherwise, the client with the second highest value is removed and so forth, until the condition is respected in *l*. In case some clients have the same value of the dissatisfaction index, that with the lowest duration of jobs is first selected to reduce the impact of the reassignment in case of equal dissatisfaction.
3. The reassignment of all clients removed from their original cluster is carried out by applying the same *k* − *NN* algorithm already employed for the initial assignments, excluding the clusters that were overloaded and from which clients were removed to avoid ending up with the previous assignments.

This procedure is repeated until all sub-problems respect the condition *r*^(*l*)^ ≤ *η*, or until there are no more clusters to move clients to. In the latter case, the decomposition is not successful due to workload violations in the clustering, though the original problem can be either feasible or infeasible.

Finally, as the procedure might leave some clusters with no clients, each caregiver in an empty cluster is moved to the closest cluster with at least one client. Thus, the number of sub-problems to solve is equal to or lower than the number |*L*| of initial clusters obtained with Γ. This final reassignment of caregivers is also performed considering the Manhattan distance over the strict preferences.

If all sub-problems provide a solution, the combination of solutions gives that of the overall problem. Otherwise, either the overall problem is infeasible or the current decomposition is unable to provide a solution. In this case, we provide the solution of the solved sub-problems, together with the percentage of jobs for which the assignment is successfully provided; repair algorithms from the VRP literature [24, 25] can then be employed to complete the solution.

**Fig. 1:**
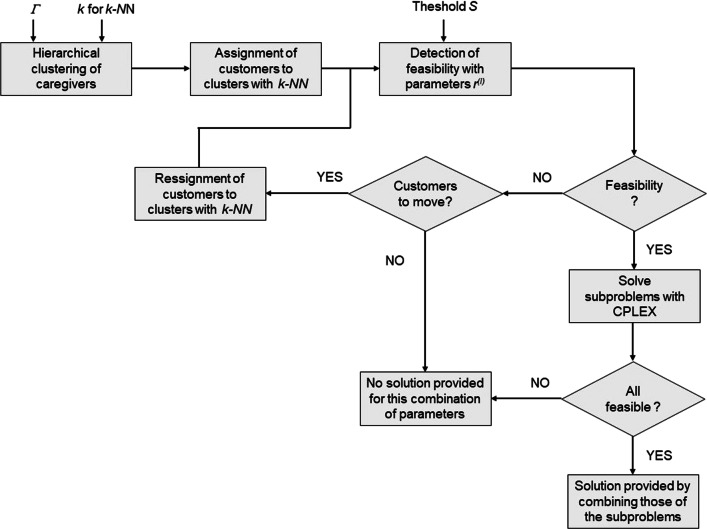
Block diagram of the cluster-based decomposition

A block diagram of the overall approach is sketched in Fig. 1.

## Case study

The approach has been validated considering realistic data obtained from the above mentioned provider that inspired our work.

Possible new clients contact the provider and, if they live in a covered area, they define the weekly pattern of requests in terms of starting time and duration for each visit. All other features regarding the visits are defined according to the job characteristics presented in Section 3.3. In particular, no job requires more than one caregiver, and for this reason this possibility was excluded in the model.

The agreed timetable is then set for a period of at least four weeks; if the client asks to change something, the changes will be effective only from the following period. Thus, the setting is quite static.

Caregivers’ homes are considered as the initial and final location for shifts. Travel times are not considered as working time, and caregivers are not paid for the time spent traveling, even when they move from one client to the next. However, the provider tries to minimize the total travel time to increase efficiency. The travel time between two locations is calculated as the average of the expected driving times in the two directions (symmetric travel times).

Client requests longer than 8 hours are split into jobs with a maximum duration *d*_*i*_ of 8 hours. According to the USA regulation, a caregiver cannot work for more than 16 hours a day. Therefore, as anticipated in the problem description, the resting period during night shifts (8 hours from 11 pm to 7 am or from 12 pm to 8 am) is not computed as working time and not paid. The regular working time of each caregiver is made up of 40 working hours per week. The hours worked beyond this threshold are considered overtime and paid more. Each caregiver is required to declare the maximum overtime he/she is willing to work; therefore, their total workload cannot exceed the regular working hours plus the maximum overtime declared.

In the current layout, as observed from data, all caregivers are willing to work on weekends (*ξ*_*k*_ = 1 ∀*k* ∈ *K*), while only about 92% of them are willing to stay overnight at a client’s home.

As is commonly done, the provider aims at minimizing the incurred overtime costs. However, as the provider introduces the idea of chargeable overtime, part of the overtime is paid by clients and is not an additional cost from the provider perspective. If a caregiver works for more than 40 hours per week with one client and this client is willing to pay overtime, the provider has no extra costs in assigning those extra hours to a single caregiver, provided that his/her maximum overtime threshold is respected. Indeed, most clients choose to pay overtime if they require more than 40 hours per week.

Finally, the provider considers the compatibility between clients and caregivers in terms of strict and soft preferences, as described in Section 3.2. Strict preferences are defined by the provider as “necessary or very important”, while soft preferences are simply considered as “preferences” that are not that relevant. In particular, 9 criteria are included in the strict preferences and 9 in the soft ones. Examples of “necessary or very important” (strict) are: female or male caregiver; caregiver with insured car and/or driving license; specific certifications as cardiopulmonary resuscitation or first aid. Examples of “preferences” (soft) are: smoker or non-smoker caregiver; caregiver accepting a smoker client; caregiver accepting dogs or cats; caregiver with good cooking skills; caregiver with a top evaluation from previous clients. Both clients and caregivers must answer a survey containing these criteria shown as yes/no questions; the clients can specify if they are interested in each item and answer the yes/no question only if interested.

At present, the provider has a pool of 37 caregivers serving 18 clients, whose needs range from two hours a week to a 24/7 live-in. Each live-in request is broken down in a series of shifts per day, including those with a night shift (i.e. with *n*_*i*_ = 1). For example, a first shift starts at 7 am and ends at 3 pm, and a second shift starts immediately after and ends at 11pm. This second shift has *n*_*i*_ = 1, i.e. the following 8 hours from 11 pm to 7 am of the following day are the unpaid resting period. A 24/7 live-in service is requested by approximately 30% of clients. However, the number of assisted clients is rapidly increasing, thus justifying the need for a planning tool.

## Computational experiments

Additional experimental tests have been conducted to evaluate the solution of the problem and the computational performance of the decomposition approach in realistic instances generated based on the considered provider. In particular, two groups of instances have been generated (*small* and *large* instances), as detailed in Section 7.1.

In all experiments, we have considered one week as the time horizon and the set *H* includes the hours within this time horizon; thus, *D* = 24 in constraints (22) and (23). According to work regulations in the considered provider, *τ* = 40, *ρ* = 8 and β = 7 in the experiments. Travel times have a 15-minute discretization, i.e. 0.25 time periods, while job durations have a 1-hour discretization, i.e. 1 time period.

Finally, the weights in the objective function have been set to reflect the decision-making strategy of the company, for which the matching of strict preferences has the highest priority. As mentioned, a lexicographic approach is considered for the multi-objective formulation. For this purpose, we have set *α*_*M*_ = 10^6^ and *α*_*F*_ = *α*_*O*_ = *α*_*T*_ = 1. The first weight is much higher than the others to perform the lexicographic approach, while the others are equal as no information is available from the company apart from the fact that weights are comparable.

The decomposition is analyzed in Section 7.2. The experimental plan has evaluated the impact of the cut-off point Γ, the neighborhood parameter *k* for the *k* − *NN*, and the threshold *η* (with *η* ≤ 1). The following levels (values) have been tested:

- Cut-off point Γ: two levels have been considered. The first level Γ_*I*_ is the lowest possible number of clusters, 1 excluded, for the instance, i.e. the number of branches after the first branching in the dendrogram. The second level Γ_*II*_ is half of the number of leaves in the dendrogram, i.e. half of the highest number of possible clusters. Higher values have been neglected as they easily make the sub-problems infeasible.
- Neighborhood parameter *k*: two levels have been considered. The first level *k*_*I*_ is always equal to 1; the second level *k*_*II*_ is equal to 30% of the number of caregivers in the biggest cluster before the reassignment of caregivers in clusters with no clients. It is worth noting that the value of *k*_*II*_ depends not only on the instance, but also on the generated clusters, i.e. on Γ.
- Threshold *η*: two values have been considered, 1 and 0.9. The former is the maximum possible value, while the latter has been chosen not far from 1 in order to keep a high quality of the solutions.

The plan has included all their combinations (with a total of 8 cases) for both small and large instances.

The impact of the decomposition parameters on the solution is further analyzed in Section 7.3 to provide guidance for their choice. The effect of chargeable overtime on the overtime paid by the provider and on the continuity of care is finally studied in Section 7.4.

All problems and sub-problems have been solved with CPLEX 12.8 on a Windows Server 2016 machine equipped with an Intel Xeon Gold 6130 processor at 2.1 GHz (with 32 cores) and 64 GB of RAM installed. A time budget of 3600 seconds (1 hour) has been assigned to each problem. When the problem is solved without any clustering, a time limit of 3600 seconds has been imposed to the solver. When the problem is divided into sub-problems, the time limit assigned to each is proportional to the ratio between the number of jobs in the cluster and the total number of jobs of the problem, with a minimum value of 300 seconds, and ensuring that the sum of time limits over the sub-problems is equal to 3600 seconds. Finally, no memory limit has been imposed.

### Tested instances

Tests have been conducted on 20 realistic instances generated based on the observed mix of clients and caregivers in the considered provider. The first 10 *small* instances have the same number of clients and caregivers of those in the real case, i.e. 18 clients and 37 caregivers. The other 10 *large* instances include 30 clients and 60 caregivers, to simulate the provider future growth while respecting the ratio between caregivers and clients in the original dataset.

More specifically, instances have been randomly sampled from the original dataset, maintaining the same features of the observed data, as summarized below. Firstly, the factors that are relevant to the problem have been identified. Clients’ factors include: strict and soft preferences, together with the related interests in expressing them; willingness to pay overtime; number of requested jobs and their initial time, duration and night shift characterization. Caregivers’ factors include: strict and soft preferences; willingness to stay overnight; willingness to work during weekends, maximum number of weekly overtime hours.

For each factor, an empirical probability distribution has been defined based on the observations in the dataset. A binomial distribution is assumed for binary parameters, whose probability of success equals the ratio of positive observations. Levels have been defined for the other parameters and the probabilities of observing values in each level have been derived from the dataset. Thus, a multinomial distribution with these probabilities has been assumed.

The parameters of each job are correlated with each other. Once the number of jobs for a client has been sampled, the other features of each job (starting time, duration and night shift characterization) have been sampled considering a hierarchical approach. Indeed, for each value of number of client’s jobs, batches of jobs that are coherent with the total number in terms of starting time, duration and night shift characterization have been defined and the probability of each batch has been derived from the data. Then, a multinomial distribution conditioned to the drawn number of jobs has been assumed with these probabilities.

With this approach, each *small* or *large* instance has been independently sampled. The generated instances together with their features are reported in Table 2. They have been ordered in increasing order of demand-to-workload ratio in the instance, which is defined as:

$$ r^{overall} = \frac{{\sum}_{i \in I} d_{i}}{{\sum}_{k \in K}{S_{k}}} $$

As for the other characteristics of the instances, they are in agreement with those described in Section 6. The instances refer to one week due to the weekly pattern of jobs. Clients with a 24/7 live-in request (about 30%) have the same request from a time perspective, though they may express different preferences. The other requests are quite different from client to client. Caregivers have *τ* = 40, with many of them available for possible overtime. All of them are willing to work on weekends while 92% are available at night. In all cases, a perfect matching of strict preferences is impossible, because no caregiver meets all requests of any client.

**Table 2:** Characteristics of the instances

Group	Instance	*r*^*overall*^	Number of jobs	Mean jobs per client	Demand (total duration of all jobs in hours)	Mean duration per job in hours (max 8 hours)	Mean percentage strict preferences requested per client	Percentage of clients willing to pay overtime
Small	1	0.464	127	7	787	6	35%	83%
	2	0.548	132	7	918	7	35%	89%
	3	0.636	153	9	1062	7	36%	44%
	4	0.657	145	8	980	7	36%	78%
	5	0.666	149	8	1049	7	36%	89%
	6	0.675	139	8	995	7	33%	78%
	7	0.681	147	8	1014	7	35%	61%
	8	0.712	166	9	1119	7	38%	67%
	9	0.741	146	8	1043	7	32%	72%
	10	0.761	158	9	1103	7	41%	67%
Large	1	0.458	200	7	1181	6	34%	77%
	2	0.619	232	8	1568	7	34%	83%
	3	0.629	237	8	1568	7	32%	60%
	4	0.658	267	9	1881	7	36%	77%
	5	0.665	262	9	1823	7	37%	80%
	6	0.676	250	8	1649	7	37%	67%
	7	0.682	247	8	1731	7	37%	77%
	8	0.744	268	9	1913	7	34%	77%
	9	0.770	277	9	1968	7	37%	70%
	10	0.850	268	9	1933	7	34%	67%

### Decomposition results

We consider in this section the reference model without the additional term (30) in the objective function.

The solutions of the problem with no cluster-based decomposition in the instances are reported in Table 3. They are first reported in terms of the overall objective function (*OF*), which is however mainly influenced by the strict preference mismatches, due to the lexicographic approach with *α*_*M*_ much higher than *α*_*F*_, *α*_*O*_ and *α*_*T*_. Then, the counts of events of different types occurred in the solution are also reported: number of mismatches for strict preferences (*m*_*M*_), number of mismatches for soft preferences (*m*_*F*_), total overtime paid by the provider *OVT*, and total caregivers’ travel time (*TT*). They are expressed in terms of counts to allow easy understanding by provider managers, as suggested by those of the considered provider. We also report the computational time taken to obtain the solution (*CT*) and the optimality gap (*Gap*), which is computed as the difference between the objective function value and the best bound provided by CPLEX, divided by the value of the objective function and expressed in percentage.

**Table 3:** Solution of the entire problem with no cluster-based decomposition: overall objective function (*OF*); mismatches of strict preferences (*m*_*M*_); mismatches of soft preferences (*m*_*F*_); total overtime paid by the provider *OVT*; total caregivers’ travel time (*TT*); computational time to get the solution (*CT*); percent optimality gap (*Gap*)

Group	Inst	Output						
	(*r*^*overall*^)	*OF*	*m*_*M*_	*m*_*F*_	*OVT*	*TT*	*CT*	*Gap*
Small	1 (0.464)	232002285.25	37	337	49	57.25	TL	1.8 ⋅ 10^− 5^%
	2 (0.548)	120002512.75	15	348	22	72.75	TL	1.1 ⋅ 10^− 5^%
	3 (0.636)	219002612.75	32	343	62	76.75	TL	1.6 ⋅ 10^− 5^%
	4 (0.657)	272002906	35	398	12	83.00	TL	7.6 ⋅ 10^− 6^%
	5 (0.666)	138003514.50	20	487	7	69.50	TL	1.7 ⋅ 10^− 5^%
	6 (0.675)	216002549.75	29	332	60	92.75	TL	1.1 ⋅ 10^− 5^%
	7 (0.681)	76003424	11	492	24	52.00	TL	2.3 ⋅ 10^− 5^%
	8 (0.712)	467003686	73	503	111	110.00	TL	3.9%
	9 (0.741)	112002832	14	396	62	55.00	TL	8.5 ⋅ 10^− 5^%
	10 (0.761)	641003625.75	85	471	98	111.75	TL	1.4 ⋅ 10^− 5^%
Large	1 (0.458)	No solution					TL	-
	2 (0.619)	No solution					TL	-
	3 (0.629)	No solution					TL	-
	4 (0.658)	No solution					TL	-
	5 (0.665)	No solution					TL	-
	6 (0.676)	No solution					TL	-
	7 (0.682)	No solution					TL	-
	8 (0.744)	No solution					TL	-
	9 (0.770)	No solution					TL	-
	10 (0.850)	No solution					TL	-

TL denotes the time limit of 3600 seconds

The results show that the problem is never solved to optimality. As for small instances, a solution is always found, whose optimality gap is lower than 10^− 4^% in 9 out of 10 cases. When this order of magnitude is obtained with *m*_*M*_≠ 0, the best matching in terms of strict preferences is found and the solution could only be improved with regards to the other terms in the objective function. On the contrary, in the small instance 8, the higher gap means that the best matching of strict preferences is not guaranteed. Finally, *m*_*M*_ is always lower than *m*_*F*_ according to the lexicographic approach. As for large instances, the time limit is always reached without providing any solution.

The outputs of the cluster-based decomposition are reported in Tables 4 and 5 for small instances, and in Tables 6 and 7 for large instances. The number of generated sub-problems (#_*subp*_) and the solution obtained over the sub-problems are listed only for the successful clusterings (combinations of Γ, *k* and *η*). We report the percentage of jobs for which a solution is provided (%_*sol*_), i.e. the percentage of jobs assigned to a sub-problem which provided a solution. All sub-problems provide a solution if %_*sol*_ = 100*%*; otherwise, some jobs are not assigned. The solution is then expressed in terms of the sum of *OF*, *m*_*M*_, *m*_*F*_, *OVT* and *TT* over the sub-problems with a solution. The sum of the computational times *CT* over the sub-problems with a solution is also reported, together with the maximum percent optimality gap with respect to the CPLEX lower bound over the sub-problem (*Gap*^*max*^). Finally, the best solution among those with %_*sol*_ = 100*%* for each instance, if any, is highlighted in bold; in case the best solution occurs for more than one combination of Γ, *k* and *η*, all rows are highlighted in bold.

**Table 4:** Cluster-based decomposition in small instances 1-5. Solutions the successful combinations of Γ, *k* and *η*: number of sub-problems (#_*subp*_); percentage of jobs for which a solution is provided over the sub-problems (%_*sol*_); sum of *OF*, *m*_*M*_, *m*_*F*_, *OVT*, *TT* over the sub-problems with a solution; sum of the computational times over them (*CT*); maximum percent optimality gap over them (*Gap*^*max*^)

Instance	Input			Overall output								
(*r*^*overall*^)	Γ	*k*	*η*	#_*subp*_	%_*sol*_	*OF*	*m*_*M*_	*m*_*F*_	*OVT*	*TT*	*CT*	*Gap*^*max*^
1 (0.464)	I(2)	I(1)	1	2	100%	316002123.25	50	321	24	55.25	3017	3.0⋅ 10^− 6^%
	II(11)	I(1)	1	8	97%	440002157.5	66	323	20	64.5	1037	2.1⋅ 10^− 6^%
	I(2)	II(9)	**1**	**1**	**100%**	**232002285.25**	**37**	**337**	**49**	**57.25**	**TL**	**1.8**⋅ 10^− 5^%
	II(11)	II(3)	1	5	93%	639001669.75	96	241	34	59.75	196	0.0%
	I(2)	I(1)	0.9	2	100%	316002121.75	53	317	19	53.75	3016	2.5⋅ 10^− 6^%
	II(11)	I(1)	0.9	8	98%	560002247.75	79	335	4	70.75	1108	1.1⋅ 10^− 6^%
	I(2)	II(9)	**0.9**	**1**	**100%**	**232002285.25**	**37**	**337**	**49**	**57.25**	**TL**	**1.8**⋅ 10^− 5^%
	II(11)	II(3)	0.9	5	93%	639001669.75	96	241	34	59.75	196	0.0%
2 (0.548)	**I(2)**	**I(1)**	**1**	**1**	**100%**	**120002512.75**	**15**	**348**	**22**	**72.75**	**TL**	**1.1**⋅ 10^− 5^%
	II(10)	I(1)	1	4	48%	1162.5	0	174	18	31.5	13	0.0%
	**I(2)**	**II(9)**	**1**	**1**	**100%**	**120002512.75**	**15**	**348**	**22**	**72.75**	**TL**	**1.1**⋅ 10^− 5^%
	II(10)	II(3)	1	5	100%	184002992.5	23	410	20	72.5	377	0.0%
	**I(2)**	**I(1)**	**0.9**	**1**	**100%**	**120002512.75**	**15**	**348**	**22**	**72.75**	**TL**	**1.1**⋅ 10^− 5^%
	II(10)	I(1)	0.9	7	100%	292002743	38	385	19	80	1264	2.1⋅ 10^− 6^%
	**I(2)**	**II(9)**	**0.9**	**1**	**100%**	**120002512.75**	**15**	**348**	**22**	**72.75**	**TL**	**1.1**⋅ 10^− 5^%
	II(10)	II(3)	0.9	6	100%	225002631.25	33	362	34	79.25	110	0.0%
3 (0.636)	I(2)	I(1)	1	2	100%	229003065	34	420	39	69	2693	2.6⋅ 10^− 5^%
	II(10)	I(1)	1	4	100%	267002942	38	420	18	75	2475	1.8⋅ 10^− 5^%
	**I(2)**	**II(7)**	**1**	**1**	**100%**	**219002612.25**	**32**	**343**	**62**	**76.25**	**TL**	**1.6**⋅ 10^− 5^%
	II(10)	II(5)	1	4	95%	279003171.5	37	416	51	66.5	2680	4.3⋅ 10^− 4^%
	I(2)	I(1)	0.9	2	100%	229003065	34	420	39	69	2693	2.6⋅ 10^− 5^%
	II(10)	I(1)	0.9	5	100%	267002957.75	38	414	43	70.75	2263	9.2⋅ 10^− 3^%
	I(2)	II(7)	0.9	2	95%	191003051.5	25	402	96	98.5	3314	7.4⋅ 10^− 5^%
	II(10)	II(5)	0.9	4	100%	272003285.25	36	455	63	64.25	2686	3.6%
4 (0.657)	I(3)	I(1)	1	3	100%	384002578.25	49	358	23	81.25	36	0.0%
	**I(3)**	**II(5)**	**1**	**3**	**100%**	**495002511.50**	**70**	**357**	**11**	**72.50**	**131**	**0.0%**
	I(3)	I(1)	0.9	3	100%	399002575	52	380	27	68	38	0.0%
	**I(3)**	**II(5)**	**0.9**	**3**	**100%**	**495002511.50**	**70**	**357**	**11**	**72.50**	**129**	**0.0%**
5 (0.666)	**I(2)**	**I(1)**	**1**	**1**	**100%**	**138003514.50**	**20**	**487**	**7**	**69.50**	**TL**	**1.7**⋅ 10^− 5^%
	II(10)	I(1)	1	4	100%	448002886.5	67	398	35	68.5	1850	1.2⋅ 10^− 5^%
	**I(2)**	**II(9)**	**1**	**1**	**100%**	**138003514.50**	**20**	**487**	**7**	**69.50**	**TL**	**1.7**⋅ 10^− 5^%
	II(10)	II(5)	1	4	100%	318003566.25	50	477	12	58.25	2004	1.8⋅ 10^− 6^%
	**I(2)**	**I(1)**	**0.9**	**1**	**100%**	**138003514.50**	**20**	**487**	**7**	**69.50**	**TL**	**1.7**⋅ 10^− 5^%
	**I(2)**	**II(9)**	**0.9**	**1**	**100%**	**138003514.50**	**20**	**487**	**7**	**69.50**	**TL**	**1.7**⋅ 10^− 5^%
	II(10)	II(5)	0.9	6	100%	615002806.5	89	380	19	84.5	1665	3.5⋅ 10^− 6^%

TL denotes the time limit of 3600 seconds, and the best solution among those with %_*sol*_ = 100*%* is highlighted in bold

**Table 5:** Cluster-based decomposition in small instances 6-10

Instance	Input			Overall output								
(*r*^*overall*^)	Γ	*k*	*η*	#_*subp*_	%_*sol*_	*OF*	*m*_*M*_	*m*_*F*_	*OVT*	*TT*	*CT*	*Gap*^*max*^
6 (0.675)	I(2)	I(1)	1	2	100%	245002553.75	35	341	56	88.75	2925	7.2⋅ 10^− 6^%
	I(2)	II(9)	1	2	100%	233002461	32	317	60	93	3329	1.2⋅ 10^− 5^%
	**I(2)**	**I(1)**	**0.9**	**2**	**100%**	**221002579.25**	**30**	**340**	**60**	**87.25**	**3322**	**9.9**⋅ 10^− 6^%
	I(2)	II(9)	0.9	2	98%	269002308.75	36	301	47	81.75	3329	8.1⋅ 10^− 6^%
7 (0.681)	I(2)	I(1)	1	2	100%	76003487.25	11	493	22	59.25	3201	2.9⋅ 10^− 5^%
	II(3)	I(1)	0.9	3	100%	280003629	40	498	28	74	2056	1.1%
	**I(2)**	**II(9)**	**1**	**1**	**100%**	**76003424.00**	**11**	**492**	**24**	**52.00**	**TL**	**2.3**⋅ 10^− 5^%
	II(3)	II(5)	0.9	3	100%	340003366.25	47	473	28	68.25	1857	1.3⋅ 10^− 1^%
8 (0.712)	I(3)	I(1)	1	3	100%	527003591.5	82	515	92	92.5	2420	6.0⋅ 10^− 5^%
	II(10)	I(1)	1	5	47%	718001190.25	119	197	22	44.25	6	0.0%
	**I(3)**	**II(5)**	**1**	**2**	**100%**	**515003613.25**	**82**	**511**	**44**	**76.25**	**2540**	3.7⋅ 10^− 5^%
	II(10)	II(5)	1	4	100%	706003697.25	112	517	37	81.25	616	0.0%
	I(3)	I(1)	0.9	3	100%	632003484	103	482	28	78	2003	1.6⋅ 10^− 6^%
	I(3)	II(5)	0.9	2	100%	582003338.25	96	475	34	70.25	2191	9.4⋅ 10^− 7^%
	II(10)	II(5)	0.9	4	100%	706003697.25	112	517	37	81.25	627	0.0%
9 (0.741)	**I(2)**	**I(1)**	**1**	**2**	**100%**	**112002809.00**	**14**	**395**	**54**	**56.00**	**3217**	**9.5**⋅ 10^− 5^%
	II(8)	I(1)	1	4	90%	88002755.25	11	366	23	53.25	2443	5.5⋅ 10^− 5^%
	I(2)	II(9)	1	1	100%	112002832	14	396	62	55	TL	8.5⋅ 10^− 5^%
	II(8)	II(5)	1	3	90%	120002434	16	326	26	50	2721	2.4⋅ 10^− 5^%
	**I(2)**	**I(1)**	**0.9**	**2**	**100%**	**112002809.00**	**14**	**395**	**54**	**56.00**	**3219**	**9.5**⋅ 10^− 5^%
	I(2)	II(9)	0.9	2	100%	232002716	30	370	48	49	3269	3.5⋅ 10^− 5^%
10 (0.761)	I(2)	I(1)	1	2	98%	689003212.75	90	405	120	115.75	3318	1.8⋅ 10^− 5^%
	I(2)	II(9)	1	2	98%	689003212	90	409	104	111	3317	1.8⋅ 10^− 5^%
	I(2)	I(1)	0.9	2	100%	792003180.75	101	427	90	83.75	3229	5.4⋅ 10^− 7^%
	**I(2)**	**II(9)**	**0.9**	**2**	**100%**	**792003179.75**	**101**	**427**	**98**	**79.75**	**3229**	**2.8**⋅ 10^− 7^%

The table has the same structure of Table 4

**Table 6:** Cluster-based decomposition in large instances 1-5

Instance	Input			Overall output								
(*r*^*overall*^)	Γ	*k*	*η*	#_*subp*_	%_*sol*_	*OF*	*m*_*M*_	*m*_*F*_	*OVT*	*TT*	*CT*	*Gap*^*max*^
1 (0.458)	I(2)	I(1)	1	2	4%	6000137	3	40	0	7	0	0.0%
	II(14)	I(1)	1	10	98%	128002727.25	22	448	98	120.25	913	7.7%
	I(2)	II(17)	1	1	0%	0	0	0	0	0	0	0.0%
	II(14)	II(7)	1	10	98%	124002280.75	26	337	90	114.75	2053	7.3%
	I(2)	I(1)	0.9	2	4%	6000137	3	40	0	7	0	0.0%
	II(14)	I(1)	0.9	10	99%	184002580.75	36	437	70	134.75	912	6.0%
	I(2)	II(17)	0.9	1	0%	0	0	0	0	0	0	0.0%
	II(14)	II(7)	0.9	10	98%	237002083.5	45	340	89	112.5	1973	5.9%
2 (0.619)	I(3)	I(1)	1	3	24%	54001206.5	11	179	21	43.5	2	0.0%
	II(15)	I(1)	1	10	100%	373004928.75	61	685	26	165.75	459	0.0%
	I(3)	II(13)	1	1	0%	0	0	0	0	0	0	0.0%
	II(15)	II(7)	1	7	96%	276003846.5	38	514	100	141.5	1772	6.4%
	I(3)	I(1)	0.9	3	24%	54001206.5	11	179	21	43.5	2	0.0%
	**II(15)**	**I(1)**	**0.9**	**8**	**100%**	**325004618.25**	**48**	**628**	**26**	**206.25**	**1381**	**3.6**⋅ 10^− 6^%
	I(3)	II(13)	0.9	1	0%	0	0	0	0	0	0	0.0%
3 (0.629)	II(12)	I(1)	1	9	91%	458004786.5	70	638	86	109.5	409	0.0%
	**I(2)**	**I(1)**	**1**	**2**	**100%**	**620002899.50**	**91**	**410**	**116**	**110.50**	**2566**	**3.6**⋅ 10^− 5^%
	I(2)	II(11)	1	2	12%	774	0	96	0	6	2	0.0%
	II(12)	II(5)	1	9	95%	509003566	78	489	137	116	584	0.0%
	II(12)	I(1)	0.9	9	100%	624004280	102	583	88	114	49	0.0%
	**I(2)**	**I(1)**	**0.9**	**2**	**100%**	**620002899.50**	**91**	**410**	**116**	**110.50**	**2580**	**3.6**⋅ 10^− 5^%
	I(2)	II(11)	0.9	2	12%	774	0	96	0	6	2	0.0%
	II(12)	II(5)	0.9	9	100%	621003804.5	93	543	118	112.5	143	0.0%
4 (0.658)	I(2)	I(1)	1	2	19%	156000831.5	20	114	14	25.5	15	0.0%
	**II(16)**	**I(1)**	**1**	**8**	**100%**	**688005849.25**	**99**	**796**	**142**	**151.25**	**1798**	**1.2**⋅ 10^− 4^%
	I(2)	II(13)	1	2	0%	0	0	0	0	0	0	0.0%
	II(16)	II(7)	1	8	100%	994005551.25	144	747	94	171.25	1509	9.7⋅ 10^− 5^%
	I(2)	I(1)	0.9	2	19%	156000831.5	20	114	14	25.5	15	0.0%
	I(2)	II(13)	0.9	2	0%	0	0	0	0	0	0	0.0%
5 (0.665)	I(2)	I(1)	1	2	100%	426005714.5	63	798	142	149.5	TL	3.0%
	II(16)	I(1)	1	11	91%	866005301.75	135	725	27	111.75	606	2.0⋅ 10^− 1^%
	I(2)	II(13)	1	2	31%	112002257.75	14	293	4	21.75	1131	2.2⋅ 10^− 6^%
	**I(2)**	**I(1)**	**0.9**	**2**	**100%**	**379006103.75**	**57**	**835**	**141**	**154.75**	**TL**	**1.3%**
	I(2)	II(13)	0.9	2	34%	112002225	14	294	4	33	1230	2.0⋅ 10^− 6^%

The table has the same structure of Table 4

**Table 7:** Cluster-based decomposition in large instances 6-10

Instance	Input			Overall output								
(*r*^*overall*^)	Γ	*k*	*η*	#_*subp*_	%_*sol*_	*OF*	*m*_*M*_	*m*_*F*_	*OVT*	*TT*	*CT*	*Gap*^*max*^
6 (0.676)	I(3)	I(1)	1	3	23%	72000980.75	10	133	28	43.75	7	0.0%
	I(3)	II(15)	1	1	0%	0	0	0	0	0	0	0.0%
	**II(16)**	**II(5)**	**1**	**7**	**100%**	**1182004367.75**	**166**	**623**	**125**	**111.75**	**1366**	2.8⋅ 10^− 6^%
	I(3)	I(1)	0.9	3	23%	72000980.75	10	133	28	43.75	7	0.0%
	I(3)	II(15)	0.9	1	0%	0	0	0	0	0	0	0.0%
7 (0.682)	**I(3)**	**I(1)**	**1**	**3**	**100%**	**862005444.75**	**117**	**740**	**150**	**128.75**	**3326**	**8.4**⋅ 10^− 1^%
	II(14)	I(1)	1	6	97%	1337004115	188	549	82	153.5	2413	2.0⋅ 10^− 5^%
	I(3)	II(11)	1	2	22%	64001180.5	8	169	4	14.5	19	0.0%
	II(14)	II(5)	1	5	100%	971005054.75	130	695	95	106.75	2639	1.3⋅ 10^− 5^%
	I(3)	I(1)	0.9	3	37%	231001873.75	30	252	19	44.75	3013	6.4⋅ 10^− 6^%
	I(3)	II(11)	0.9	2	22%	64001180.5	8	169	4	14.5	20	0.0%
8 (0.744)	I(2)	I(1)	1	2	21%	15001188.25	3	166	11	31.25	20	0.0%
	I(2)	II(13)	1	2	0%	0	0	0	0	0	0	0.0%
	I(2)	I(1)	0.9	2	18%	15001413.5	3	180	17	19.5	13	0.0%
	I(2)	II(13)	0.9	2	12%	56000733.5	7	95	4	13.5	2	0.0%
9 (0.770)	I(2)	I(1)	1	2	10%	925	0	116	0	5	5	0.0%
	I(2)	II(13)	1	2	5%	457.5	0	56	0	1.5	1	0.0%
	I(2)	I(1)	0.9	2	16%	248001153	34	151	4	17	20	0.0%
	I(2)	II(13)	0.9	2	19%	528001104	76	151	16	28	26	0.0%
10 (0.850)	I(2)	I(1)	1	2	10%	84000478.75	11	54	16	22.75	2	0.0%
	I(2)	II(17)	1	1	0%	0	0	0	0	0	0	0.0%
	I(2)	I(1)	0.9	2	8%	64000715.75	9	86	0	11.75	1	0.0%
	I(2)	II(17)	0.9	2	6%	60000440	9	57	0	12	1	0.0%

The table has the same structure of Table 4

Most gaps are lower than 10⋅ 10^− 4^%, and a higher gap is generally observed when *m*_*M*_ = 0 in the corresponding sub-problem. It is worth remarking that this is not immediately visible from the tables, as they report the maximum gap and the sum of *m*_*M*_ over the sub-problems. In particular, the gap is lower than 10⋅ 10^− 4^% when *m*_*M*_ = 0 in all sub-problems but 2 (of large instances). Therefore, these gaps show that both the entire problem (only for small instances) and the sub-problems are generally solved to optimality with respect to the first term of the objective function, and that the optimality gap is due to the other terms, for which a better solution could be obtained while keeping the same value for the first term.

In small instances, for which a solution of the problem with no cluster-based decomposition is possible (Table 3), the decomposition performed worse, but only in 4 out of 10 instances, where the objective functions are 182% (in instance 4), 102% (in instance 6), 110% (in instance 8) and 124% (in instance 10) of the corresponding solution for the entire problem with no decomposition, respectively. Moreover, in 2 out of 10 instances, the solution from the decomposition is slightly better than the corresponding solution with no decomposition, i.e., the difference between the objective value function with and without decomposition is equal to -0.50 and -23.00 in instances 3 and 9, respectively. Though we are aware of the solution detriment that could be associated with the decomposition, it is often limited.

In large instances, the decomposition approach allows us to provide a solution with %_*sol*_ = 100 in 6 out of 10 instances, where a solution for 100% of jobs is provided within the time limit for at least one combination of Γ, *k* and *η*. Moreover, we have clearly observed that the decomposition approach does not provide a solution to the entire problem for higher *r*^*overall*^ values, i.e., in instances 8 (with *r*^*overall*^ = 0.744), 9 (with *r*^*overall*^ = 0.770) and 10 (with *r*^*overall*^ = 0.850). Actually, a solution for the entire problem is not provided also in instance 1, where however %_*sol*_ = 99*%* indicates a solution for almost all jobs. We may argue that the decomposition is not able to find a solution for all jobs where the entire problem could be infeasible in itself. As a matter of facts, in these instances we found similar %_*sol*_ under the different decomposition layouts, which could indicate that these percentages are driven by the instance instead of the decomposition. We cannot formally prove that, as we did not find a solution for the entire problem (see Table 3), but our results indicate this motivation.

We can observe that *m*_*M*_ is always much less than *m*_*F*_, as in Table 3, confirming that the primary goal is to minimize the mismatches of strict preferences. As for small instances, we observe that, the value of *m*_*M*_ either increases or remains constant with the decomposition. Considering the best solutions in bold, the worst increase is observed in instance 4 where the value of *m*_*M*_ has doubled. This is anyway a limited increase, in agreement with the structure of the decomposition, that creates clusters based on the matching of strict preferences. We can therefore hypothesize that the values of *m*_*M*_ in large instances are not too deteriorated compared to their optimal value. As for the other metrics *m*_*F*_, *OVT* and *TT*, they have increased or decreased based on the instance. In particular, in instance 4 where *m*_*M*_ has doubled, all these metrics have decreased. Thus, rather than deteriorating the overall solution quality, the decomposition seems to select solutions that favor specific terms in the objective function with respect to the hierarchy assigned by the weights *α*_*M*_, *α*_*F*_, *α*_*O*_ and *α*_*T*_.

### Analysis of decomposition parameters

In this section we evaluate the impact of the decomposition parameters Γ, *k* and *η* on the solution, based on the results in Tables 4-7, and provide guidance for their choice.

We may first observe that the quality of the solution deteriorates somewhat when #_*subp*_ increases; however, if the value of #_*subp*_ is too small, we do not reach %_*sol*_ = 100*%*, especially in large instances.

To quantify the impact of the parameters, we have conducted an independent ANOVA for *%*_*sol*_ and for the metrics *m*_*M*_, *m*_*F*_, *OVT* and *TT*. All the ANOVAs consider the decomposition parameters as factors with two levels. As for *%*_*sol*_, observations refer to all the combinations of parameters in each instance; they are taken from Tables 4-7 in the case of combinations with a solution, while *%*_*sol*_ = 0 is assumed for the others as no solution is provided. As for the metrics, only the observations in Tables 4-7 are taken and, to normalize them with respect to the percentage of solution provided, the values are divided by such percentage. The ANOVA outputs, obtained with *R*, are reported in Table 8.

**Table 8:** *p*-values of the terms in the ANOVA for the different variables

Term	Metric				
	%_*sol*_	*m*_*M*_	*m*_*F*_	*OVT*	*TT*
(a) Small					
Γ	0.029 *	0.060 ∙	0.741	0.062 ∙	0.672
*k*	0.499	0.955	0.881	0.352	0.699
*η*	0.524	0.683	0.997	0.804	0.687
Γ × *k*	0.485	0.889	0.853	0.966	0.614
Γ × *η*	0.756	0.712	0.386	0.859	0.238
*k* × *η*	0.826	0.600	0.642	0.355	0.673
(b) Large					
Γ	0.042 *	0.349	0.004 **	0.006 **	0.140
*k*	0.057 ∙	0.966	0.900	0.072 ∙	0.001 ***
*η*	0.050 *	0.406	0.756	0.386	0.412
Γ × *k*	0.158	0.793	0.144	0.001 ***	0.086 ∙
Γ× *η*	0.070 ∙	0.124	0.464	0.946	1.000
*k* × *η*	0.961	0.228	0.425	0.297	0.282

Reported significance levels are as follows: “***” for 0.001; “**” for 0.01; “*” for 0.05; “∙” for 0.1

The results show that only Γ significantly affects the solution in small instances, both in terms of %_*sol*_ and some metrics. In these cases, Γ_*II*_ is associated with a reduction of %_*sol*_, an increase of *m*_*M*_ and a reduction of *OVT* with respect to Γ_*I*_ Thus, Γ_*I*_ is recommended to obtain a solution for several jobs associated with lower *m*_*M*_ values.

In large instances, both Γ and *k*, as well as their interaction, are significant for several metrics. In the significant cases, Γ_*II*_ is associated with a reduction of %_*sol*_, *m*_*F*_ and *OVT* with respect to Γ_*I*_; *k*_*II*_ is associated with a reduction of %_*sol*_, *OVT* and *TT* with respect to *k*_*I*_; Γ_*II*_ × *k*_*II*_ shows an increase of *OVT* and *TT*. Thus, Γ_*I*_ and *k*_*I*_ are recommended in order to obtain a solution for the highest possible number of jobs, while no recommendation is possible regarding the quality of the solution, as *m*_*M*_ is not significantly affected and the other secondary terms show alternate effects. Also *η* significantly affects %_*sol*_, with an increase of *η*_1_ with respect to *η*_0.9_ and for *k*_*II*_ × *η*_1_.

From the practical viewpoint, these analyses indicate that some combinations of decomposition parameters perform better than others. Thus, when our approach has to be applied in practice, we suggest to repeat the decomposition with different parameters starting from the better combinations identified by the ANOVA; if the result from a decomposition setting is not satisfactory, a new decomposition can be performed considering other promising combinations.

### Impact on overtime and continuity of care

In this section we analyze the impact of chargeable overtime on the overtime paid by the provider and the continuity of care. To this end, together with *OVT*, we consider the number of caregivers assigned to each client willing to pay overtime (denoted by *nk*^*ϕ*= 1^) and to each client unwilling to pay overtime (denoted by *nk*^*ϕ*= 0^), both expressed in terms of mean value and standard deviation over the corresponding clients.

We compare *OVT*, *nk*^*ϕ*= 1^ and *nk*^*ϕ*= 0^ under three configurations: the reference configuration already tested in Section 7.2; the configuration in which the continuity of care is reinforced through the additional term (30) in the objective function and the related constraints; a configuration in which all clients are unwilling to pay overtime (forcing *ϕ*_*c*_ = 0 ∀*c* ∈ *C*). In the last configuration, to effectively compare the alternatives, *nk*^*ϕ*= 1^ and *nk*^*ϕ*= 0^ are computed while keeping the same division of the original instance, though all clients are unwilling to pay overtime. The second configuration is run with *α*^∗^ = 10^5^ to give a high priority to the additional term, but lower than the priority of strict preference mismatching. Moreover, due to the slower convergence observed in this configuration, its time limit has been tripled.

We focus on small instances for which a solution without decomposition is obtained and on a couple of large instances for which *#*_*subp*_ = 2 and *%*_*sol*_ = 100*%*, i.e., on instances 3 and 5 decomposed with Γ_*I*_, *k*_*I*_ and *η* = 0.9.

Results are reported in Table 9. They show that *OVT* values are always lower with chargeable overtime than without, being less than half in several cases. When comparing the first two alternatives, there is not a clear trend as *OVT* is lower under the first or the second alternative depending on the instance. Thus, chargeable overtime is clearly effective in unburdening the provider while the effectiveness of the additional term (30) depends on the instance. As for the continuity of care, it is not adequately pushed by the reference formulation when compared to the case without chargeable overtime. In fact, when comparing the first and the third alternative, *nk*^*ϕ*= 1^ and *nk*^*ϕ*= 0^ values are almost similar. This is due to the presence non-consecutive jobs from the same client and a high percentage of clients with *ϕ*_*c*_ = 1 (whose values are reported in Table 9). The additional term (30) pushes the continuity of care with lower *nk*^*ϕ*= 1^ values in some instances, while it is not effective in others. More specifically, it is effective for lower *r*^*overall*^ values because it exploits the flexibility of the instance, for which alternative solutions are available due to the margin between demand and capacity. When *r*^*overall*^ increases, this margin reduced and the problem becomes more complex. Accordingly, the additional term (30) is associated with a non-negligible optimality gap even under longer computational times. Thus, aiming at providing a solution in a limited computational time from the practical viewpoint, such a term should be included only if there is margin between the demand and the capacity. Otherwise, it could be counterproductive.

**Table 9:** Values of *OVT*, *nk*^*ϕ*= 1^ and *nk*^*ϕ*= 0^ in the small instances without decomposition and in large instances 3 and 5 decomposed with Γ_*I*_, *k*_*I*_ and *η* = 0.9, for which *#*_*subp*_ = 2 and *%*_*sol*_ = 100*%*: reference configuration with chargeable overtime; configuration in which the continuity of care is reinforced through (30); configuration in which all clients are unwilling to pay overtime

Group	Inst	*r*^*overall*^	Percentage		With chargeable		With chargeable overtime and			Without chargeable		
			of *ϕ*_*c*_ = 1		overtime (reference)		(30) in objective function			overtime (*ϕ*_*c*_ = 0∀*c* ∈ *C*)		
				*OVT*	*nk*^*ϕ*= 1^	*nk*^*ϕ*= 0^	*OVT*	*nk*^*ϕ*= 1^	*nk*^*ϕ*= 0^	*OVT*	*nk*^*ϕ*= 1^	*nk*^*ϕ*= 0^
Small	1	0.464	83.3%	49	2.5 ± 1.2	2.7 ± 2.1	67	2.3 ± 1.5	3.0 ± 2.6	137	2.5 ± 1.2	3.0 ± 2.6
	2	0.548	88.9%	22	2.5 ± 1.2	3.5 ± 3.5	26	1.8 ± 0.9	4.5 ± 4.9	93	2.6 ± 1.5	3.0 ± 2.8
	3	0.636	44.4%	62	3.5 ± 1.7	2.4 ± 0.8	57	2.3 ± 1.0	2.2 ± 1.1	116	4.0 ± 1.7	2.4 ± 0.8
	4	0.657	77.8%	12	2.6 ± 1.2	2.0 ± 1.4	32	1.9 ± 1.0	2.3 ± 1.9	84	2.7 ± 1.3	2.0 ± 1.4
	5	0.666	88.9%	7	3.3 ± 1.2	1.0 ± 0.0	69	2.8 ± 1.2	1.0 ± 0.0	129	3.3 ± 1.1	1.0 ± 0.0
	6	0.675	77.8%	60	2.9 ± 1.5	2.3 ± 0.5	52	2.3 ± 1.0	2.5 ± 0.6	146	2.7 ± 1.4	2.3 ± 0.5
	7	0.681	61.1%	24	2.5 ± 0.9	2.9 ± 1.9	52	2.0 ± 1.0	2.7 ± 2.1	93	3.0 ± 0.9	3.0 ± 1.8
	8	0.712	66.7%	111	3.9 ± 1.9	2.7 ± 2.4	207	4.4 ± 2.4	3.5 ± 1.6	247	3.8 ± 1.9	2.2 ± 1.8
	9	0.741	72.2%	62	3.2 ± 1.6	2.8 ± 1.5	16	2.1 ± 0.9	3.4 ± 1.5	132	3.5 ± 1.8	3.2 ± 1.8
	10	0.761	66.7%	98	2.9 ± 1.1	5.3 ± 3.1	139	2.9 ± 1.3	5.0 ± 3.1	188	2.7 ± 1.0	5.0 ± 2.8
Large	3	0.619	60.0%	116	2.6 ± 1.2	2.7 ± 0.9	103	1.2 ± 0.9	2.8 ± 2.2	162	2.9 ± 1.4	2.7 ± 0.8
	5	0.665	80.0%	141	3.7 ± 2.2	3.5 ± 2.2	102	2.3 ± 1.4	3.3 ± 1.9	200	3.0 ± 1.4	3.2 ± 1.8

## Conclusion

We have formulated a novel HC scheduling problem and proposed a cluster-based decomposition algorithm to obtain solutions for larger instances. The chargeable overtime has been introduced for the first time in the literature. Combined with the needs of the real provider operating in the USA, it represents an advancement for the HC practice. Indeed, this idea offers more flexibility to HC providers that want to increase the provided service level without excessively increasing overtime cost, giving clients the possibility to further increase the service level by allowing the payment of the difference in cost. In our opinion, this idea is immediately applicable to other providers and our work can be a useful tool to apply it.

We have also proposed a cluster-based decomposition to address real-life instances of the problem, which proved effective in our tests. Moreover, the analysis of the outcomes suggested to perform the decomposition paying attention to Γ and *k* in the case of large instances, preferring Γ_*I*_ and *k*_*I*_.

Results also showed that, in this specific provider, the reference model is able to reduce the overtime paid by the provider, while non-consecutive jobs from the same client and the high percentage of clients with *ϕ*_*c*_ = 1 require the additional term in the objective function to adequately pursue continuity of care, which is effective when the solution converges to the optimal one.

The ongoing work will be dedicated to the development of heuristic, metaheuristic or matheuristic approaches to provide approximate solutions for both the problem or the sub-problems. On the one hand, the idea is to completely avoid decomposition; on the other hand, it is to complete the solution when the percentage of jobs for which a solution is provided is less than 100%. In particular, variable neighborhood search strategies will be considered to develop fast algorithms for large instances.

An assessment of the feasibility of the overall problem without providing a solution could also be implemented. This will allow to determine whether infeasibilities (%_*sol*_ < 100*%*) are due to the solution approach or to the instance itself.

Finally, we will consider other approaches to tackle the multi-criteria problem other than the lexicographic one considered in this work; for example, we will consider a threshold method or the Pareto frontier analysis.
